# Response (minimum clinically relevant change) in ASD symptoms after an intervention according to CARS-2: consensus from an expert elicitation procedure

**DOI:** 10.1007/s00787-021-01772-z

**Published:** 2021-04-07

**Authors:** Lucie Jurek, Matias Baltazar, Sheffali Gulati, Neda Novakovic, María Núñez, Jeremy Oakley, Anthony O’Hagan

**Affiliations:** 1 Child and Adolescent Psychiatry Department, Center for Assessment and Diagnostic of Autism, Le Vinatier Hospital, Bron, France; 2 Centre of Excellence and Advanced Research on Childhood Neurodevelopmental Disorders, Child Neurology Division, Department of Pediatrics, All India Institute of Medical Sciences, New Delhi, India; 3 Day Care Centre for Children and Adolescents with Developmental Disabilities, Belgade, Serbia; 4 Faculty of Psychology, Universidad Autónoma de Madrid, Campus de Cantoblanco, Madrid, Spain; 5 School of Mathematics and Statistics, The University of Sheffield, Hounsfield Road, Sheffield, UK

**Keywords:** Autism Spectrum Disorder, Measure, Change, CARS, Response, Expert knowledge elicitation, SHELF

## Abstract

The lack of consensual measures to monitor core change in Autism Spectrum Disorder (ASD) or response to interventions leads to difficulty to prove intervention efficacy on ASD core symptoms. There are no universally accepted outcome measures developed for measuring changes in core symptoms. However, the CARS (Childhood Autism Rating Scale) is one of the outcomes recommended in the EMA Guideline on the clinical development of medicinal products for the treatment of ASD. Unfortunately, there is currently no consensus on the response definition for CARS among individuals with ASD. The aim of this elicitation process was to determine an appropriate definition of a response on the CARS2 scale for interventions in patients with Autism Spectrum Disorder (ASD). An elicitation process was conducted following the Sheffield Elicitation Framework (SHELF). Five experts in the field of ASD and two experts in expert knowledge elicitation participated in an 1-day elicitation workshop. Experts in ASD were previously trained in the SHELF elicitation process and received a dossier of scientific evidence concerning the topic. The response definition was set as the mean clinically relevant improvement averaged over all patients, levels of functioning, age groups and clinicians. Based on the scientific evidence and expert judgment, a normal probability distribution was agreed to represent the state of knowledge of this response with expected value 4.03 and standard deviation 0.664. Considering the remaining uncertainty of the estimation and the available literature, a CARS-2 improvement of 4.5 points has been defined as a threshold to conclude to a response after an intervention. A CARS-2 improvement of 4.5 points could be used to evaluate interventions' meaningfulness in indivudals. This initial finding represents an important new benchmark and may aid decision makers in evaluating the efficacy of interventions in ASD.

**Supplementary Information:**

The online version contains supplementary material available at 10.1007/s00787-021-01772-z.

## Introduction

Autism Spectrum Disorder (ASD) is a common neurodevelopmental disorder associated with social impairments and repetitive behavior and interests [1]. Intervention for ASD has been the focus of intensive research over recent years [2, 3]. With the increase of ASD prevalence, it has become extremely important to develop appropriate biomedical, behavioral and developmental treatments [4]. Advance in this field remains nevertheless complicated, one major hindrance being the lack of consensual measures to monitor core change in ASD or response to interventions which lead to difficulty to prove intervention efficacy on ASD core symptoms [2, 4, 5].

Efficacy claims of an intervention require not only statistical significance but also clinical meaningfulness. One proposed approach to address this question is a responder analysis, in which a continuous primary efficacy measure is dichotomized into "responders" and "non-responders." Such responder classifications help in interpreting data clinically and speak directly to the question of fundamental interest in clinical science and practice: “Is this therapy benefitting the patient? “. Responder definitions are based on a threshold of changes in endpoint scores and are defined as a magnitude of change that is considered important to the patient.

If experts emphasize the need to develop psychometrically sound outcome measures [5, 6], it is also well reported that the proliferation of a scattered variety of instruments to assess changes in specific symptoms or abilities prevent effective comparisons across intervention studies and the development of best practice recommendations [2, 3, 5]. That is why the core symptoms of ASD represent obvious outcome measurement targets [6] and should, if possible, be assessed with one or few tools. It is also important to be able to monitor progress in a way that is not only reliable and systematic but also practical and time-efficient for families, schools and other service providers. It needs to be accessible in different countries (translated in different languages) to allow the use of common assessments across locations [5]. It is also important to have a tool that suits for individuals from various age ranges in order to be able to conduct reliable follow-up studies (e.g. following a cohort of adolescent patients as they reach adulthood)[2].

Very few tools suit all these criteria. The CARS-2 was chosen in our study as it is a well-established, widely used measure with good psychometric properties, recommended by the European Medicines Agency for the development of medicinal products for ASD [3, 7–9]. Designed to inform autism diagnosis with the use of cut-off scores, it also allows to quantify the severity of the disorder, which makes it useful also for outcome evaluation [8, 10]. It contains 15 items scored from 1 (no symptom) to 4 (severe symptom) in 0.5 intervals. Two versions are available: the standard version (CARS-ST) and the “high functioning” version (CARS-HF), adapted for verbally fluent individuals older than 6 with an intellectual quotient greater than 80. For the CARS-ST, a total score of 15–29.5 is in favor of the absence of ASD diagnosis; a score of 30–36.5 reflects mild to moderate autism; a score of 37–60 reflects moderate to severe autism. For the CARS-HF, cut-off scores are defined as 15–27.5 for the absence of ASD, 28–33.5 for mild to moderate ASD and 34–60 for moderate to severe ASD. The tool has been translated into multiple languages and psychometrics properties have been evaluated in a broad age range (from early childhood to mid-adulthood), allowing for its use for individuals aged 2 years old and up [8].

Despite its widespread use, little is known about the clinical relevance of the CARS-2 total score and no consensus exists on the minimal change in CARS-2 total score which should reflect an individual clinically significant improvement. Most of the time clinicians must rely on subjective experience with individual patients and populations to interpret CARS-2 scores and the clinical significance of various degrees of change. In research setting, guidelines for the development of pharmacological treatment recommends to complete primary analyses by responder analyses using pre-specified criteria for response. As there is currently no consensus on the response definition for CARS among individuals with ASD, an elicitation workshop was organized to obtain a balanced scientific assessment of the response definition for CARS, based on the evidence.

Opinion-seeking methods, such as expert knowledge elicitation, allow the consideration of both clinically important and realistic difference [11]. Expert Knowledge Elicitation is the formulation of the expert’s knowledge in the form of a probability distribution. Elicitation is typically a dialogue between experts and a facilitator. The facilitator is knowledgeable in probability and statistics and in how to conduct the dialogue in such a way as to elicit the expert’s knowledge as faithfully and with as less influence as possible [12]. The experts are selected based on their knowledge about the quantity to be determined. Elicitation involves subjective judgment, and it is, therefore, important for the exercise to be conducted according to a rigorous, well-designed protocol, in order to obtain scientifically valid judgments [17].

In order to evaluate an appropriate definition of a response on the CARS2 scale for interventions in patients with Autism Spectrum Disorder (ASD), an expert knowledge elicitation process was conducted using the Sheffield Elicitation Framework (SHELF), one of the leading protocols in the field [13].

## Method

### Expert selection/participants

Experts were selected to participate to the elicitation task, according to the following criteria: (a) experts were all familiar with ASD as a result of extensive clinical and/or research practice; (b) they all had a background in conducting research in the field of ASD; (c) experts should collectively cover knowledge regarding various age groups and intellectual functioning in ASD individuals; (d) experts should be familiar with CARS-2 scale for clinical or research use.

In addition to these mandatory criteria, it was considered that having an international board (4 different countries) and experts with different backgrounds would be of interest to cover all perspectives and insights. The experts did not know each other before the process and were selected mainly upon their publications or established clinical practice.

The expert panel (listed at the end of this article) included two children and adolescent psychiatrists, two doctors in psychology, and one Special Educator and Rehabilitator, all of whom were involved in academic research and/or were clinically active. No unrepresented areas of expertise were identified. The experts thought that, collectively, their expertise and experience covered a suitable range of patient groups. No expert had a personal or financial interest regarding the elicitation outcome. A summary of the experts’ qualification can be found in Supplementary Material (S1).

Two expert knowledge elicitation facilitators (J.O., T.O.) were present to animate the workshop, one to facilitate the elicitation process and one to record key details of the elicitation session and run the software to fit probability distributions to the values elicited from the experts.

### Literature review/evidence dossier

An evidence dossier was circulated prior to the elicitation workshop (Supplementary Material, S2). This dossier provided summaries of published evidence on CARS-2 score changes that were interpreted as being clinically relevant in clinical trials [9, 14–17]. Such evidence was scarce and very few studies were published at the time of this elicitation procedure. Our result will be discussed in regard of these studies in the discussion part.

The experts also completed a training course to review the CARS-2 rating rules upon the CARS manual description for both versions of the scale (standard and high functioning). Quotation of two clinical cases was also required to evaluate inter-judge fidelity among the experts. This also served to ensure that the experts had a common understanding of the scale, thereby avoiding misunderstanding during the workshop.

### Elicitation process

- Shelf

**Fig. 1:**
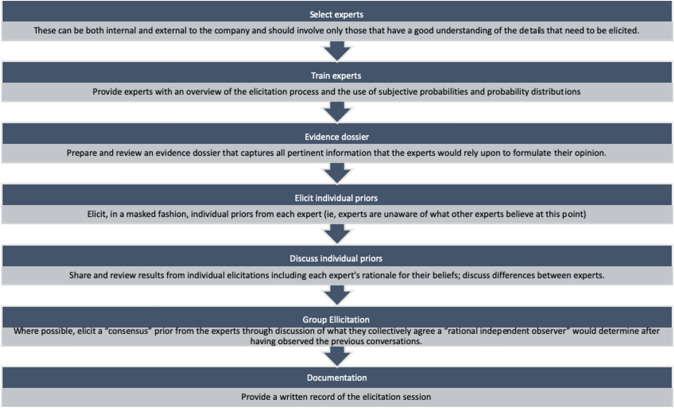
Main steps in SHELF elicitation process

Sheffield Elicitation Framework (SHELF) was used to evaluate the definition of a response (also called mean minimum clinically relevant change during the elicitation process) in CARS2 score (Fig. 1). SHELF is a package of documents, templates*** and software to carry out elicitation of probability distributions for uncertain quantities from a group of experts. The SHELF templates serve the dual role of determining a protocol to minimize cognitive biases and documenting the elicitation exercise, for a complete review see [18].

- Elicitation training

All experts took an online elicitation training course prior to the workshop, to familiarize themselves with the kinds of judgments that they would be required to make in an elicitation workshop and with how to make those judgments.

At the start of the workshop, the experts completed a training example on a subject not related to the subject of interest. This served to refresh their online training and to introduce them to the procedure for sharing, discussing and resolving their individual judgments.

- Definition of the quantity of interest (QOI)

The quantity to determine was the minimum clinically relevant change in CARS2 score. In accordance with the SHELF procedure, the definition of the Quantity of Interest is important. It must be clear to all experts, unambiguous and such that it has a unique (but unknown) value. The starting point for this exercise was that a response should be the minimum change in CARS2 score that would represent a clinically relevant benefit a subject with ASD. It was noted, however, that the minimal improvement that would be regarded as clinically relevant will vary from one patient to another, depending on the nature of the patient’s condition, their age and the CARS2 items in which the improvement arose. Also, a difference that is judged clinically meaningful by one clinician might not be by another. Because of this variation between patients and clinicians/investigators the quantity of interest was defined as a mean, averaged over patients, levels of functioning, age groups and clinicians.

The definition of this quantity was discussed by the experts prior to the elicitation process to assess whether to define the quantity of interest as an absolute change, or as a relative change. Regarding a scale not including a zero rating for the absence of symptoms and a relatively narrow range for scoring (from 1 to 4), giving a too heavy relative weight to absent symptoms compared to present significant ones, it was agreed that absolute changes were easier to interpret. Also, the use of a relative change score was deemed problematic, since it assumes that the change will not be interpreted the same way depending on the starting score. For example, an improvement of 5 points could lead to a lower relative change for participants with high severity of ASD, compared to the same improvement in participants with milder symptoms. For these two main reasons, all experts agreed to consider absolute changes only.

The variable that was elicited was the mean absolute numerical change in total CARS2 raw score that would be judged by an experienced clinician to represent a minimum clinically relevant improvement for a given patient with ASD, averaged across all patients, all age groups, all levels of functioning and all clinicians.

- Process

According to the SHELF procedure, multiple steps were made in the following order: individual elicitation with the tertile method, fitting observation, group discussion, group elicitation, feedback and discussion, choice of distribution and discussion [18].

For the group elicitation, SHELF uses behavioral aggregation where experts are asked to agree on a final probability distribution representing what a rational impartial observer (RIO) might believe after reading the evidence dossier, seeing their experts’ individual judgments and hearing their subsequent discussion [18]. The concept of RIO was explained to the experts as a hypothetical person who has been standing in the room, listening carefully to all the opinions and all the arguments that the experts have given in support of their opinions. RIO is supposed to be familiar with the elicitation topic, and so able to understand and evaluate those opinions and arguments. It would be unrealistic to imagine that the experts could agree completely about likely values of the QOI, even after extensive discussion. They would surely leave the workshop with opinions that might have been influenced by the discussion but which are nevertheless not totally in consensus. Instead, they are asked to reach agreement on what a rational impartial observer might believe. RIO would not be expected to agree exactly with any one expert, but would give some weight to each expert’s opinions. By contemplating RIO’s perspective, the experts are encouraged also to evaluate and give weight to the judgments of their fellow experts. Experience with the SHELF protocol, confirmed in the present exercise, suggests that this is an effective way for experts to reach a meaningful consensus.

Furthermore, the RIO perspective accords well with the scientific objectives of an expert knowledge elicitation. The objective is generally not to learn what any individual expert thinks, nor even to have some average opinion of the selected expert group; it is to have a representation of what a rational person would believe having studied and debated the relevant evidence.

### Statistical analysis

Statistical analysis were conducted live during the elicitation meeting by Jeremy Oakley, using the R package SHELF running on R [19, 20]. This process allowed for all experts to visualize the QOI distribution on graphs, based on their estimations, and to dynamically revise their judgments when necessary.

## Results

### General characteristics

The elicitation workshop took place in Paris, France, on March 11th, 2020 and lasted for 1 day. Due to travel restrictions during this period due to Covid-19 pandemic, two experts and the elicitation’s recorder attended the meeting remotely by videoconferencing. For the results section, experts will be denoted by letters A, B, C, D, E, and will be referred to using feminine pronouns. The facilitator will be denoted by Z and referred to using masculine pronouns.

### Individual elicitation

**Fig. 2:**
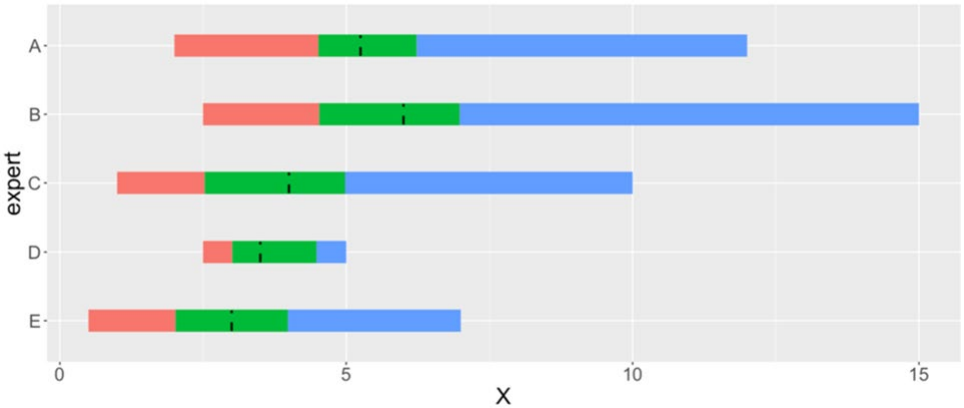
Individual elicited judgments from each expert on minimum clinically important change on CARS-2. The three colored sections represent parameter ranges judged to be equally likely by each expert. The dashed lines represent the experts’ medians. X represents the number of points on the CARS scale

Expert were asked to remember that the true value of the QOI is unknown, and, therefore, each of them would have uncertainty that could be represented as a probability distribution. Each expert would have her own opinions about the mean clinically relevant improvement and, therefore, her own probability distribution. They were prompted by the facilitator to make a series of judgments to characterize the distribution. The lower limit was defined as the minimum plausible value, below which the expert should judge if it would be very unlikely or implausible to find our QOI and vice versa for the upper plausible limit. The expert’s median was defined as the value for which the QOI was equally likely to be above or below. Finally, the expert’s upper and lower tertiles similarly divided the range of plausible values into three equally likely parts. These values were written by each expert individually, without any group discussion, before being communicated to the facilitator. They constituted individual elicitations, providing grounds for group discussion, before moving to the final stage of SHELF where experts provide a group consensus on the QOI probability function (see below). Expert values are presented in Fig. 2.

### Group discussion

The definition of the quantity of interest caused some difficulty for the experts. Z prompted some clarification from the experts, in order for them to reach a consensus on the parameters of the QOI. Several points were made: First, the quantity refers to the minimum improvement clinicians/investigators would *want* to see in patients; the minimum improvement required for a treatment to be considered as benefitting a patient. The expected change, hoped-for change or target change due to any particular treatment *was not deemed relevant* to this elicitation exercise. Second, the quantity of interest refers to a *minimum clinically relevant* change. It was made clear that this is different from a *significant* change in the statistical sense (e.g. with a p value falling under a preset threshold in a clinical trial), such as those already reported in the literature. Z also pointed out that the QOI is defined as an average (mean) clinically relevant improvement, averaged over all patients and clinicians. If, for example, the QOI were to equal 10 points, that would imply that for about 50% of patients, clinicians/investigators would require an improvement of *at least* 10 points before judging the patients to have had a benefit from treatment.

There is likely to be some variability between measurements taken on the same patient: variability between different observers assigning CARS2 scores, and/or variability in scores taken at different times on the same patient (even with no intervention in between). Small improvements in scores consistent with such variability might be dismissed as random noise.

The experts noted these points, with experts A and B in particular revising their upper limits downwards. Expert D, noting that the quantity of interest is defined in terms of the *minimum* required improvement, thought that her upper limit of 5 was still appropriate.

There was general agreement that, in a majority of patients, an improvement of 5 points would be always judged as clinically relevant. In other words, all experts agreed on the fact that there is no plausible situation in which an increase of 5 points on the scale would *not* reflect a meaningful improvement. Hence, it was very unlikely that the mean minimum required improvement could be greater than 5.

To reach a consensus, the group discussed the results presented in the evidence dossier. There was some discussion of the comparison between CGI-I and CARS2 provided on page 4 of the evidence dossier (*See supplemental material S2*). Here it was noted that (a) the standard deviation of CARS2 scores in the CGI-I responder group was relatively large, and (b) that the CARS2 estimates referred to mean changes in observed CARS2 scores, not minimum required changes in CARS2 scores. Hence it was judged difficult to make inferences from these data.

There was some discussion of Table 3 in the evidence dossier (*See supplemental material S2*) and that 17.4% of patients in the placebo group achieved an improvement of 4 points or more: was this informative for the amount of ‘background noise’ in CARS scores? One expert noted that, over the course of a study involving children and adolescents, some patients might improve, even in the placebo group; these patients may simply improve as they became older or might benefit from a placebo effect (significant improvement through mind–body self-healing processes). Hence an improvement of 4 points would not necessarily be dismissed as noise.

There was agreement that an improvement of a single point would be very unlikely to be judged relevant; the mean would need to be greater than 1.

### Group elicitation

Then the group of experts was asked to collectively determine key summaries of the probability distribution of the QOI, based on the previous discussion and with as much impartiality and rationality as possible (using the rational impartial observer framework, see “Methods” section). The probability that the QOI was inferior or equal to 3.5 was judged to be equal to 0.33. The probability that the QOI was superior to 4.75 was judged to be equal to 0.2. The probability that the QOI was inferior or equal to 4 was judged to be equal to 0.45.

The final step is to obtain a probability distribution for the QOI to reflect the available knowledge from the perspective of the rational impartial observer. The experts’ elicited group judgments are the starting point m and this step begins with the recorder fitting one or more standard probability distributions to the elicited probabilities. The group judgments are difficult for the experts to make, and at best they will be approximate. At the fitting stage they will often be varied, under the guidance of the facilitator and the recorder, to obtain a probability distribution that more accurately represents the experts’ knowledge and beliefs.

**Fig. 3:**
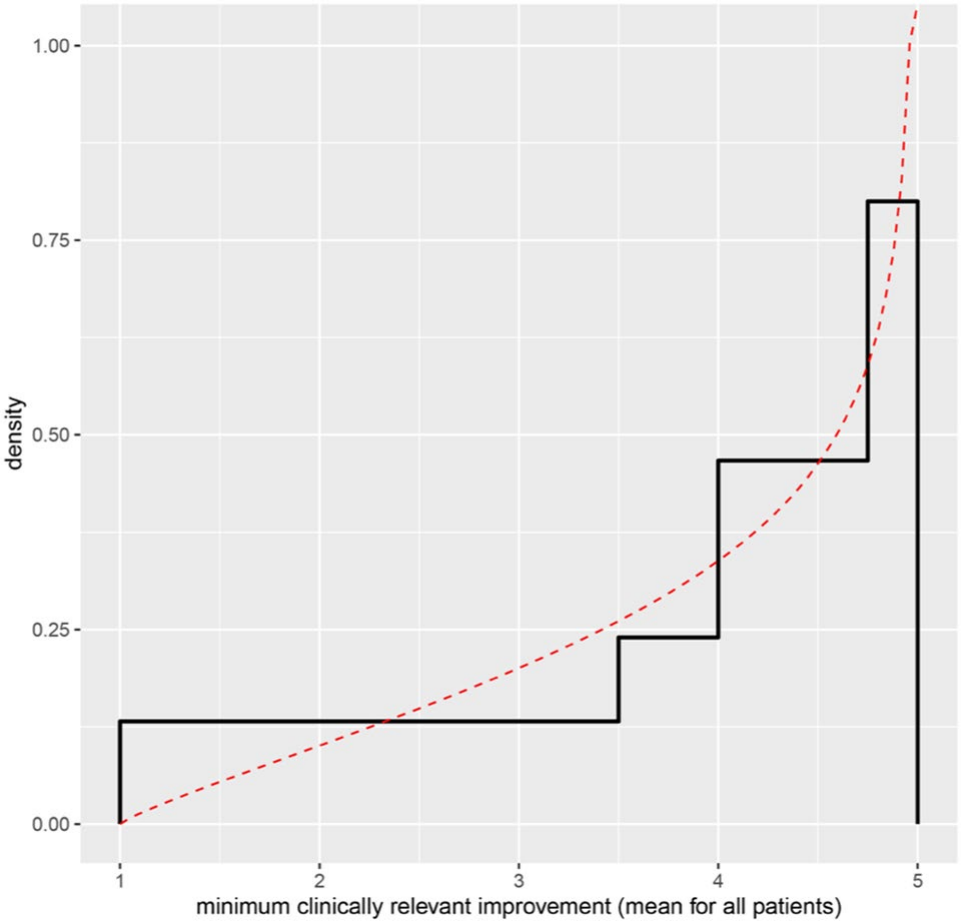
Histogram and fitted beta distribution depicting the experts’ initial group judgments

To begin this process, the experts were shown their RIO judgments in the form of a histogram and a fitted probability distribution. These are depicted in Fig. 3. In discussion with the facilitator, the experts agreed that this did not reflect their opinions; in particular, it seemed to give too much probability to low values of the QOI, particularly values below 1, and an unrealistically high probability in the range 4.75–5. It was felt that 5 should not be a hard upper limit.

The recorder showed the experts how the histogram and fitted distribution would change if the limits and probabilities were varied, and a number of alternative fits were discussed. Finally, the experts chose:

- to increase the lower plausible limit to2 and the upper plausible limit to 6,
- to reduce the first probability to P (X <  = 3.5) = 0.2,
- to reduce the second probability to P (X > 4.75) = 0.15,
- and to increase the third probability to P (X <  = 4) = 0.5.

**Fig. 4:**
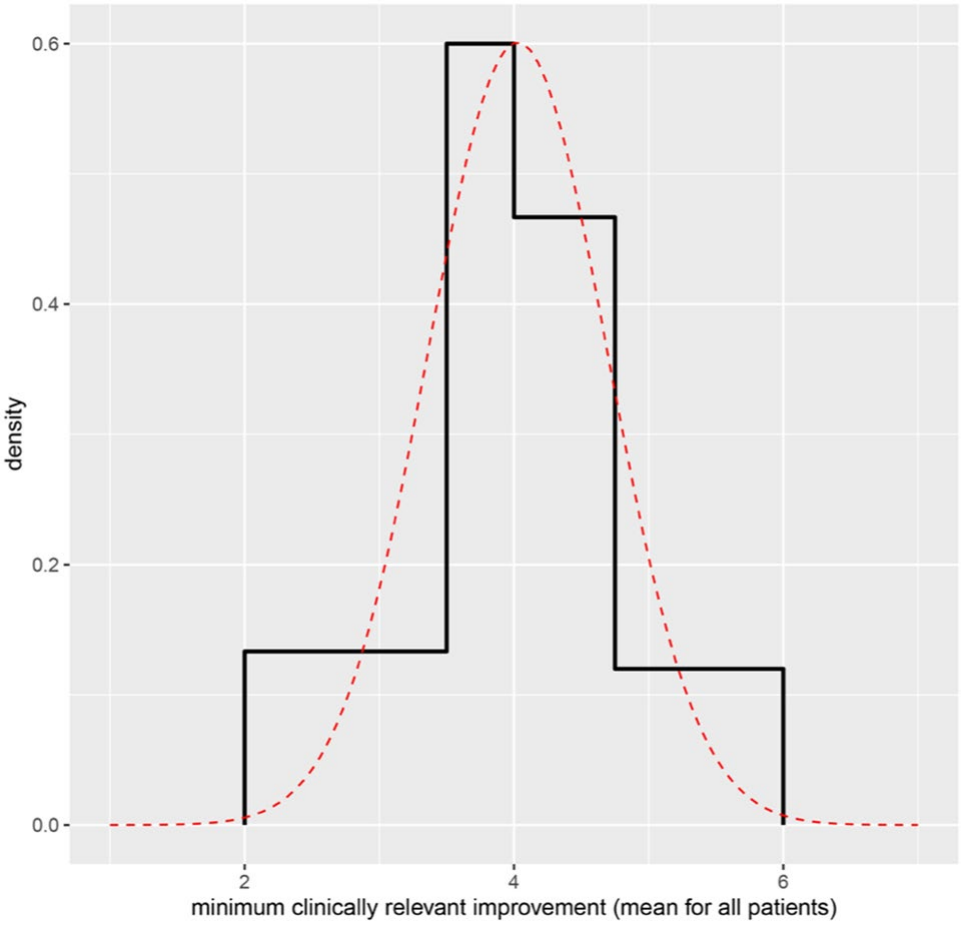
The experts’ final judgments and agreed distribution

The final distribution is shown in Fig. 4. In the chosen distribution, the fitted 90th percentile was 4.88, and there was a 7% probability of the QOI exceeding 5. The bulk of the probability was in the range 3.5–4.5.

The experts agreed that these features were appropriate and were in accordance with what a rational impartial observer would believe, based upon the evidence and the points raised during the discussion. The fitted distribution was confirmed as the outcome of the elicitation.

### Final result: CARS-2 response distribution

A normal distribution with a mean equal to 4.03 and standard deviation 0.664 was agreed to represent the available knowledge and evidence regarding the mean absolute numerical change in total CARS2 raw score that would be judged by an experienced clinician to represent a minimum clinically relevant improvement for a given patient with ASD, averaged across all patients, all age groups, all levels of functioning and all clinicians (Fig. 4).

## Discussion

Measuring change in studies pertaining to ASD is a particular difficulty in the field. CARS-2 is a widely used scale, with good psychometric properties, focusing on ASD core symptoms, corresponding to European guideline on the clinical development of medicinal products for the treatment of ASD [9]. Using an expert elicitation process, the mean minimum clinically important change on CARS-2 score was determined, to help defining a threshold to identify patients that are responders to an intervention. Our results show that on average, clinicians would like to see a 4-point improvement, as a minimal change, to conclude to a meaningful improvement after an intervention in the context of ASD.

*Coherence with other studies*

Our result is coherent with available literature on this topic (Table 1). In their study on the use of secretin in ASD, Chez et al. described that a 6-point improvement in ratings is indicative of a « clinically significant» change. Their explanation relied on the fact that by virtue of the instrument’s design, a change of 6 points in the total score leads to a change in severity classifications (e.g. from “mild to moderate autistic symptoms” to “minimal to no symptoms”) [14]. We proposed a 2-point lower score change, with the objective to define a “Minimum” Clinically Important change. Using a reliable change index method [21], Coniglio et al. obtained the exact same results as ours [15]. This index method corrects for unreliability of measurement and establishes a standard amount of change that is needed for any individual case across two repeated measures to be considered either clinically improved, unchanged, or deteriorated. Two other studies presented a target improvement for their sample size calculation. One considered a mean difference of 4 points in change from baseline in the CARS2 total score [16]. The second considered an improvement of at least 20% from baseline total score [17].

**Table 1:** Proposition of what a minimum clinically significant improvement could be on CARS-2 in available scientific literature

Authors	Study	Change definition	Score	Explanation
Chez et al	Study assessing clinical efficacy following injection of secretin via alterations in the behavioral characteristics of children with ASD	Clinically significant change	6 points on total score	Change of severity classification (from moderate to non-autistic)
Coniglio et al	RCT assessing the efficacy of a single injection of intravenous secretin on socialization and/or communication skills in children with ASD	Clinically significant improvement	4.07 points	Use of reliable change index
Lemonnier et al	RCT evaluating the effect of bumetamide on neurobehavioral function in children and adolescent with ASD	Change used to calculate SS	4 points	Calculation based on the assumption that the standard deviation for change in CARS was 4.5
Nagaraj et al	RCT to assess the efficacy and safety of risperidone in young children with ASD	Change used to calculate SS	20% improvement	Chosen after a pilot run to test the scales and after comparing the results from previous published studies in which the margin was generally found to be within this range
Our study		Minimum clinically relevant change	Mean of 4.03 points (SD 0.664)	Expert Knowledge Elicitation

*RCT *randomized controlled trial,* ASD *autism spectrum disorder,* SS *Sample size

Lemonnier et al. also described a correlation between CARS-2 and CGI-I. No calculation was done to correct the mean score at CARS-2 when CGI was reported as “minimally improved” but a graph presented the results with a median improvement around 4 points on the CARS corresponding to the “minimally improved” category [16].

*Choice of a threshold*

We could, therefore, recommend that a 4-point CARS2 improvement could be used as the definition of a response outcome in studies of interventions in ASD. In view of the remaining uncertainty about the elicited QOI, we might also prefer to suggest choosing a 4.5-point improvement, because according to the fitted distribution, we can be 70% sure that the QOI is below 4.5. This threshold is also coherent with available literature and is a more cautious choice for regulators.

*Importance of this result for randomized controlled trials*

Our study is the first one to propose a threshold for a minimum clinically relevant improvement on a scale assessing core symptoms of ASD. This threshold could be used to assess whether a subject with ASD could be considered as a responder or not regarding a treatment.

Concerning our methods, expert elicitation process has been recommended mostly when the outcome is particularly complex, which is the case in the context of ASD core symptoms [22]. Other methods, such as distribution-based approaches, rely on statistical characteristics to determine important changes in the clinical outcome [21, 22]. However, they largely ignore the main objective, which is to define the clinical importance of a given change in outcome scores, separate from their statistical significance. On the other hand, anchor-based approaches work by relating the outcome of interest to another recognized measure of clinical change: the anchor. However, the lack of studies using both CARS and an external criterion that could be considered as an anchor prevents from using this approach.

Compared to all these approaches, the expert elicitation has the advantages to account for the clinical expertise to estimate the quantity of interest. It can be used to directly elicit a clinically important change in an outcome of interest, without the need to use another instrument as a comparator. The SHELF protocol enables a clear and transparent way to elicit subjective, informed and rational ratings. Wording of the initial question was clear and specific; the background diversity of the selected experts allowed for a complete coverage on the topic and all the experts agreed with the final result.

### Limitations

Some difficulties were noted with the definition of the quantity of interest; it was particularly difficult for the experts to consider an average minimum clinically relevant improvement across all patients. Ideally, a threshold for a minimum clinically relevant improvement might be elicited for different ASD subgroups (e.g. high functioning and low functioning). Nevertheless, as most of the RCT in ASD considered a broad range of participants, a mean minimum clinically relevant improvement for all type of ASD participants is important to be used as a target change to define a response to treatment. As described in the CARS-2 manual, the same method of rating is used for the two versions of CARS-2 (CARS2-ST, CARS2-HF) and the internal structure of the two versions and the original version of the CARS are similar. The experts collectively agreed to define the quantity of interest as the mean clinically relevant improvement across all patients and all versions [8]. For future studies, it would, therefore, be interesting to evaluate each version separately, with the use of an anchor-based or distribution-based method to complete the results the present study.

The SHELF protocol uses a behavioral approach to resolve the experts’ judgments into a single distribution. The behavioral approach has the difficulty of persuading experts with differing opinions to reach “consensus”. Such procedure could be impacted by personality issues (e.g. introvert experts being less willing to provide their opinion compared to extroverts, etc.) or status issues (junior versus senior expert). The facilitator’s role is to manage the experts and to address possible sources of bias in group interactions. The presence of an experienced facilitator in our study was designed to avoid unbalanced weighting of arguments in the discussion within the experts.

## Conclusion

Our results provide a threshold for the minimum clinically relevant improvement on the CARS-2 that can be used to determine patients responders to interventions for ASD. An improvement of 4.5 points on CARS-2 represents a minimum clinically relevant change that can be used to classify the patient as responder.

As Authorities’ guidelines for assessing ASD in clinical trial context recommends that endpoint on key efficacy measures should be supported by responder analyses using pre-specified criteria for response, the findings are of great use for further clinical trial targeting the core symptoms in ASD.

This initial finding represents an important new benchmark and may aid decision makers in evaluating the efficiency of interventions in ASD.

## Supplementary Information

Below is the link to the electronic supplementary material.

Supplementary file1 (DOCX 450 KB)

## References

[1] American Psychiatric Association, editor (2013) Diagnostic and statistical manual of mental disorders: DSM-5. 5. ed. Washington, DC: American Psychiatric Publishing

[2] Magiati I, Moss J, Yates R, Charman T, Howlin P (2011). Is the autism treatment evaluation checklist a useful tool for monitoring progress in children with autism spectrum disorders?: Is the autism treatment evaluation checklist useful?. J Intellect Disabil Res.

[3] Provenzani U, Fusar-Poli L, Brondino N, Damiani S, Vercesi M, Meyer N (2020). What are we targeting when we treat autism spectrum disorder? A systematic review of 406 clinical trials. Autism.

[4] Bolte EE, Diehl JJ (2013). Measurement tools and target symptoms/skills used to assess treatment response for individuals with autism spectrum disorder. J Autism Dev Disord.

[5] Cunningham AB (2012). Measuring change in social interaction skills of young children with autism. J Autism Dev Disord.

[6] Lecavalier L, Bodfish J, Harrop C, Whitten A, Jones D, Pritchett J (2020). Development of the behavioral inflexibility scale for children with autism spectrum disorder and other developmental disabilities. Autism Res.

[7] McConachie H, Parr JR, Glod M, Hanratty J, Livingstone N, Oono IP (2015). Systematic review of tools to measure outcomes for young children with autism spectrum disorder. Health Technol Assess.

[8] Schopler E, Van Bourgondien ME, Wellman GJ, Love SR (2010) Childhood autism rating scale, second edition. Western psychology services

[9] European Medecine Agency (2017) Guideline on the clinical development of medicinal products for the treatment of Autism Spectrum Disorder (ASD)

[10] Geier DA, Kern JK, Geier MR (2013). A comparison of the autism treatment evaluation checklist (ATEC) and the childhood autism rating scale (CARS) for the quantitative evaluation of autism. J Ment Health Res Intellect Disabil.

[11] Fayers PM, Cuschieri A, Fielding J, Craven J, Uscinska B, Freedman LS (2000). Sample size calculation for clinical trials: the impact of clinician beliefs. Br J Cancer.

[12] O’Hagan T (2005). Elicitation. Significance.

[13] Oakley JE, O’Hagan T (2019) SHELF: the Sheffield Elicitation Framework (Version 4)

[14] Chez MG, Buchanan CP, Bagan BT, Hammer MS, McCarthy KS, Ovrutskaya I (1999). 007 Secretin and autism: A two part clinical investigation. Eur J PaediatrNeurol.

[15] Coniglio SJ, Lewis JD, Lang C, Burns TG, Subhani-Siddique R, Weintraub A (2001). A randomized, double-blind, placebo-controlled trial of single-dose intravenous secretin as treatment for children with autism. J Pediatr.

[16] Lemonnier E, Villeneuve N, Sonie S, Serret S, Rosier A, Roue M (2017). Effects of bumetanide on neurobehavioral function in children and adolescents with autism spectrum disorders. Transl Psychiatry.

[17] Nagaraj R, Singhi P, Malhi P (2006). Risperidone in children with autism: randomized, placebo-controlled, double-blind study. J Child Neurol.

[18] O’Hagan A (2019). Expert Knowledge Elicitation: Subjective but Scientific. Am Stat.

[19] Oakley J (2020) SHELF: Tools to Support the Sheffield. Elicitation Framework. R package

[20] R Core Team (2020) R: A language and environment for statistical computing. Vienna, Austria.: R Foundation for Statistical Computing

[21] Jacobson NS, Truax P (1991). Clinical significance: A statistical approach to defining meaningful change in psychotherapy research. J Consult ClinPsychol.

[22] Cook J, Hislop J, Adewuyi T, Harrild K, Altman D, Ramsay C (2014). Assessing methods to specify the target difference for a randomised controlled trial: DELTA (Difference ELicitation in TriAls) review. Health Technol Assess.

