# Supplementary material for: Response (minimum clinically relevant change) in ASD symptoms after an intervention according to CARS-2: consensus from an expert elicitation procedure
Source: Eur Child Adolesc Psychiatry. 2021 Apr 7;31(8):1–10. doi: 10.1007/s00787-021-01772-z (PMC8024930; doi:10.1007/s00787-021-01772-z)
Supplement: Supplementary file 1 — Supplementary file1 (DOCX 450 KB) [file 787_2021_1772_MOESM1_ESM.docx]

**EVIDENCE DOSSIER – CARS Responders**

# Context

There are currently no pharmacological treatments approved to manage the persistent deficits in social communication and interaction, restricted interests, and stereotyped and repetitive behaviors associated with autism spectrum disorder (ASD).

There are no universally accepted outcome measures developed for measuring changes in core symptoms from treatment. However CARS scale is one of the outcome recommended on EMA Guideline on the clinical development of medicinal products for the treatment of Autism Spectrum Disorder (ASD) EMA/CHMP/598082/2013 (June 2018). This guidance represents the main reference document available for the development of therapy in ASD.

This guideline recommends also that primary analyses of change from baseline to endpoint on key efficacy measures should be supported by responder analyses using pre-specified criteria for response.

Unfortunately there is currently no consensus on the response definition for CARS among individuals with ASD. We are organizing an elicitation workshop to obtain a balanced scientific assessment of the response definition for CARS, based on the evidence.

# The Quantity of Interest

Defining the response to treatment is needed to assist clinicians, patients and regulatory agencies in understanding the effectiveness of treatments.

Responder definitions are based on a threshold of changes in endpoint scores based on psychometric evidence and are defined as a magnitude of change that is considered important to the patient.

Responder definitions could be based on:

- The absolute change between inclusion and 6 months on the CARS2 total raw score. Let’s imagine a patient with a CARS2 total score at inclusion of 36 and after 6 months of treatment a CARS2 total score of 33. The absolute change is of 3 points (36-33).
- The relative change between inclusion and 6 months on the CARS2 total raw score. With the same patient with a CARS2 total score at inclusion of 36 and after 6 months of treatment a CARS2 total score of 33, the relative change is of 8.3% ((36-33)/36 *100=8.3%).

The quantity of interest (QoI) is defined as the average of all possible values considered as response threshold by the whole community of experts in autism. The uncertainty around this QoI corresponds to the variation in the experts’ definition of a response.

# Description of the CARS

The Childhood Autism Rating Scale, Second Edition (CARS2) is 15-item observation-based rating system designed to assist in diagnostic assessment and intervention for autism spectrum disorders (Schopler et al. 2010).

Two observational rating forms are available: the CARS2-ST (Standard Version) and the CARS2-HF (High Functioning).

- CARS2-ST: should be used for assessing individuals with overall IQs <=79, who have notably impaired communication or who are younger than 6 years of age regardless of their estimated IQ.
- CARS2-HF: should be used to assess individuals with overall IQs scores >=80, who have relatively good verbal skills and who are aged 6 or older (Schopler et al. 2010).

The CARS2 measures 15 core deficit behaviors associated with autism spectrum disorders including social-emotional understanding, expression and regulation of emotion, relationship to people, imitation, body use, object use, adaptation to change, visual response, listening response, sensory use (taste, touch, smell), fear and anxiety, verbal communication, nonverbal communication, activity level, consistency of intellectual response, and clinical impressions.

Rating values for each of the 15 CARS2 items range from 1 to 4. Generally:

- A rating value of 1 indicates that an individual’s behaviour is within normal limits for an individual of that age.
- A value of 2 means that the individual’s behavior is mildly abnormal compared with a person of the same age.
- A value of 3 indicates that the individual’s behavior is moderately abnormal for that age.
- A value of 4 indicates that the individual’s behavior is severely abnormal for someone of that age.

In addition to these four ratings, the midpoints between them (1.5, 2.5, 3.5) should be used when the behavior appears to fall between two categories.

In the framework of the current phase III studies, specific rating conventions have been implemented in order to minimize the inter-rater variability:

- A midpoint should be given in case of hesitation between 2 scores (i.e 2.5 if rater hesitates between 2 and 3)
- The highest score should be given in case of hesitation between 2 mid points (i.e 3 if rater hesitates between 2.5 & 3)

**Interpretation of CARS2-ST and CARS2-HF scores (Schopler et al. 2010)**


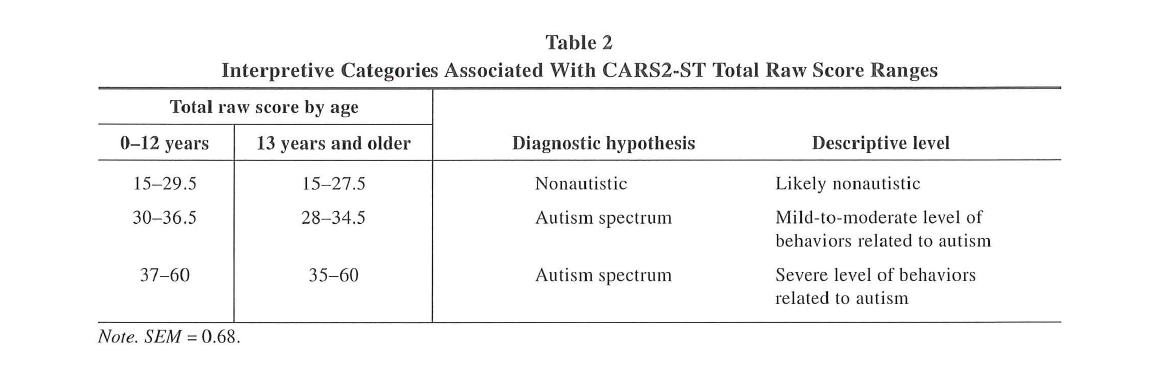


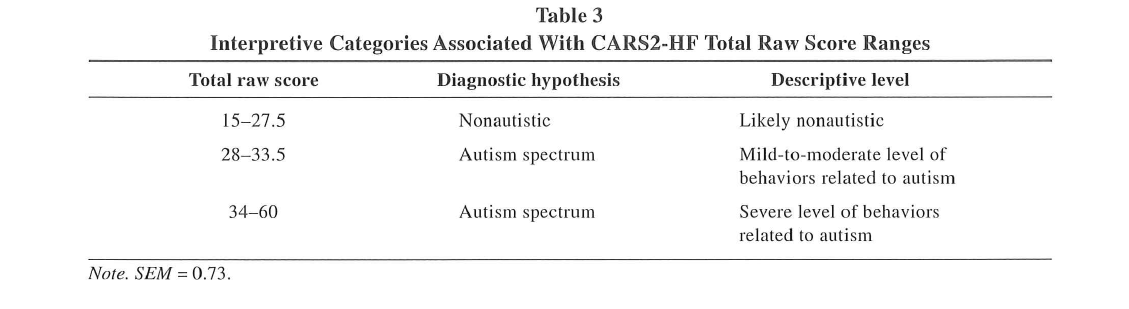


# Data available

- **Phase II clinical trial with Bumetanide in ASD children (Lemonnier et al. 2017)**

The NeuroClin02 study was a multicenter, randomized, double-blind, parallel-group, placebo-controlled, 3-month dose-ranging study to evaluate Bumetanide 0.5 mg/mL solution (0.5-1.0-2.0 mg twice daily) with the primary end point of change in CARS at Day 90 in children and adolescents (2-18 years old) with ASD.

The main objectives were to determine the optimal dose strength of Bumetanide for the pivotal Phase III study.

The CARS results are provided in the following table:

Primary endpoint: change in CARS total score from screening to day 90 in the completers

|  |  | Bumetanide  0.5 mg BID (N=20) | Bumetanide  1.0 mg BID (N=23) | Bumetanide  2.0 mg BID (N=22) | Placebo (N=23) |
| --- | --- | --- | --- | --- | --- |
| *Descriptive Statistics* | | | | | |
| Screening | N | 20 | 23 | 22 | 23 |
|  | Mean ± SD | 42.45±(4.18 | 41.13±6.01 | 41.30±5.44 | 40.41±4.89 |
|  | Median | 43.75 | 39.50 | 40.75 | 39.00 |
|  | Min ; Max | [35.0, 49.0] | [34.5, 52.0] | [34.5, 52.0] | [34.5, 52.0] |
|  |  |  |  |  |  |
| Day 90 | N | 20 | 19 | 13 | 21 |
|  | Mean ±SD | 37.48 ± 5.59 | 37.00 ± 5.31 | 37.73 ± 7.14 | 38.62 ± 4.60 |
|  | Median | 37.75 | 36.00 | 38.00 | 37.50 |
|  | Min ; Max | [26.5, 49.0] | [27.0, 48.5] | [27.0, 50.5] | [30.0, 47.5] |
|  |  |  |  |  |  |
| D90(*) - SCREENING | N | 20 | 19 | 13 | 21 |
|  | Mean ± SD | -4.98 ± 4.33 | -3.74 ± 3.28 | -5.35 ± 3.88 | -1.79 ± 2.39 |
|  | Median | -5.0 | -4.0 | -6.0 | -1.0 |
|  | Q1 ; Q3 | -9.0 ; -1.25 | -7.0 ; -1.0 | -6.5 ; -5.5 | -3.5 ; 0.0 |
|  | Min ; Max | -11.0 ; 3.0 | -10.0 ; 1.0 | -12.0 ; 3.0 | -8.5 ; 2.0 |
| *Statistical analysis* | | | | | |
| General linear model | E (SE) (1) | -2.93 (1.09) | -1.90 (1.09) | -3.22 (1.24) |  |
|  | 95% CI (2) | [-5.57 ; -0.29] | [-4.55 ; 0.73] | [-6.22 ; -0.22] |  |
|  | p-value (3) | **0.026** | 0.211 | **0.032** |  |
| *A reduction in CARS corresponds to an improvement.*  *(1) Estimate (Standard Error) of the difference between adjusted treatment group means : Bumetanide dose minus Placebo.*  *(2) Two-sided 95% Adjusted Confidence Interval of the estimate (with Dunnett-based adjustment).*  *(3) Two-sided Adjusted p-value (adjustement according to the procedure of Dunnett-Hsu for multiplicity adjustment).* | | | | | |

Responder analysis:

Despite consensus from experts, responders’ analysis using different thresholds were performed. The results are presented in the following table:


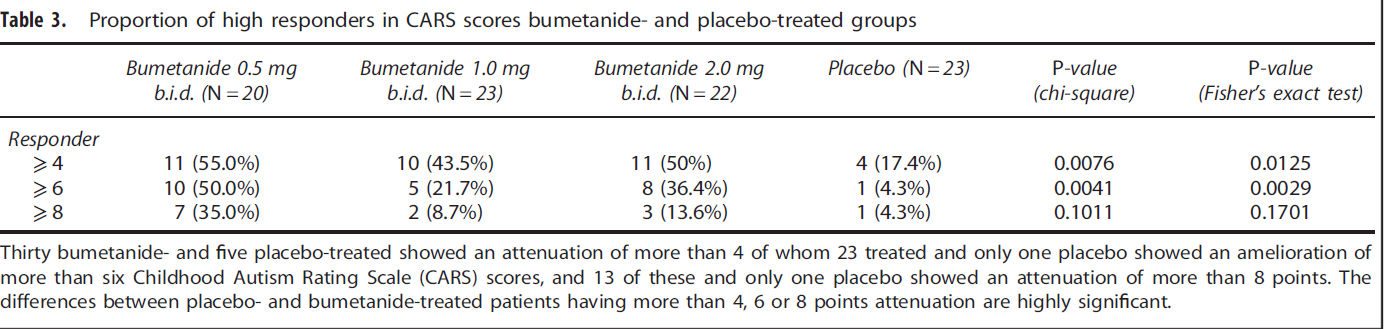


Ability to detect change (post-hoc analysis not published yet):

The Clinical GIobal Impression Improvement (CGI-I), a well-known measure of the global assessment, was used as a secondary endpoint. For this endpoint, the definition of responders is well established and corresponds to subjects with CGI-I scores of “minimally improved” or better.

Sensitivity to change (baseline to day 90) was assessed by comparing mean scores on CARS2 total score between subjects with CGI-I scores of “minimally improved” or better versus “no change” or worse all doses combined using analysis of covariance.

The results are the following:

|  | **n/N** | Change in CARS2 total score from screening to D90 in completers:  LS Mean (95% CI) |
| --- | --- | --- |
| Responders CGI-I at D90 | 53/72 | -4.78 (-5.65 ; -3.92) |
| Non responders CGI-I at D90 | 19/72 | -0.76 (-2.19 ; 0.68 ) |
| Difference between means (95% CI)* |  | -4.03 (-5.71;-2.34) |
| p-value |  | <0.001 |

**Analysis of covariance. Adjusted for baseline CARS. LS, least squares*

- **Clinical trial with Risperidone in ASD children (Nagaraj et al. 2005)**

A randomized, double-blind, placebo-controlled trial with 40 children with autism, whose ages ranged from 2 to 9 years was conducted to determine whether the use of risperidone improved functioning, especially with regard to aggressiveness, irritability, hyperactivity, social responsiveness, emotional interaction, and communication abilities based on scores assigned on the Childhood Autism Rating Scale (CARS).

Twelve of 19 children (63%) demonstrated an **improvement of at least 20% from baseline** scores in the risperidone group (median score 39.5 at baseline [range 32.5-46] to 32.00 at the end of treatment [range 24.5-40.5]), whereas none of the children in the placebo group recorded such a magnitude of improvement (median score 38.5 at baseline [range 31.5-43] to 37.5 at the end of treatment [range 30-42.5]; p-value < .001).

- **Secretin and Autism: A Two-Part Clinical Investigation (Chez et al. 2000)**

The study is an open-label study. Fifty-six children (49 boys, 7 girls, mean age =6.4 years, SD = 2.7) were enrolled in the first part of the study. All children received two IU of secretin per kilogram of body weight intravenously over a period of 1 to 2 minutes. CARS were completed by parents at baseline and post injection visits. Average duration of follow-up was 3.7 weeks (SD = 1.4).

The response definition reported in this publication is the following:

“We designated a **6-point improvement** in ratings as indicative of a “clinically significant” change because, by virtue of the instrument’s design, a change of 6 points or more reflects a change in diagnostic classifications in children who fall in the midrange “moderately autistic” category. Such a change may be considered a liberal definition of improvement since it represents only a 10–15% improvement over baseline.”

- **A randomized, double-blind, placebo-controlled trial of single-dose intravenous secretin as treatment for children with autism (Coniglio et al. 2001)**

Sixty subjects with autism were randomly selected and assigned to either treatment or placebo group. Subjects in the treatment group received 2.0 clinical units of secretin per kilogram of body weight as a single intravenous dose. Subjects in the placebo group received normal saline solution. Neurodevelopmental and behavioral assessments were performed for all subjects before injection and at 3 and 6 weeks after injection.

The response definition reported in this publication is the following:

“The reliable change index (Jacobsen et al. 1991) was used to develop a metric for determining clinically significant improvement on the CARS from intake to 3 weeks and from intake to 6 weeks. This index corrects for unreliability of measurement and establishes a standard amount of change that is needed for any individual case across 2 repeated measures to be considered clinically improved, unchanged, or deteriorated. When this index is used for the CARS total score, **a decrease of 4.07 points or greater** results in a case being classified as clinically improved, and an increase of 4.07 points or greater results in a case being classified as deteriorated.”

**Summary of the evidence**

|  | | | |
| --- | --- | --- | --- |
| **Reference** | **CARS Responder definitions** | | |
| Lemonnier et al. 2017 | Improvement >= 4 points | Improvement >= 6 points | Improvement >= 8 points |
| Chez et al. 2000 | Improvement >= 6 points | Improvement of 10-15% |  |
| Nagaraj et al. 2005 | Improvement of 20% |  |  |
| Coniglio et al. 2001 | Improvement >= 4.07 points |  |  |

**References**

Jacobsen N, Truax P. Clinical significance: a statistical approach to defining meaningful change in psychotherapy research. J Consult Clin Psychol 1991;59:12-9.

Michael G. Chez, Cathleen P. Buchanan, Bradley T. Bagan,1 Michael S. Hammer, Karla S. McCarthy, Irina Ovrutskaya, Caralynn V. Nowinski, and Zamia S. Cohen. Secretin and Autism: A Two-Part Clinical Investigation. Journal of Autism and Developmental Disorders, Vol. 30, No. 2, 2000

Guideline on the clinical development of medicinal products for the treatment of Autism Spectrum Disorder (ASD). EAM/CHMP/598082/2013

Lemonnier E, Villeneuve N, Sonie S, Serret S, Rosier A, Roue M, Brosset P, Viellard M, Bernoux D, Rondeau S, Thummler S, Ravel D, Ben-Ari Y. Effects of bumetanide on neurobehavioral function in children and adolescents with autism spectrum disorders. Transl. Psychiatry 7:e1056. 2017 PE0140375

Ravishankar Nagaraj, MD; Pratibha Singhi, MD; Prahbhjot Malhi, PhD. Risperidone in Children With Autism: Randomized, Placebo-Controlled, Double-Blind Study. Journal of Child Neurology / Volume 21, Number 6, June 2006

Schopler E, Van Bourgondien ME, Wellman GJ, Love SR. Childhood Autism Rating Scale, Second Edition (CARS2). In: Childhood Autism Rating Scale, Second Edition (CARS2). Western Pychological Services. 2010. PE0154244

Susan J. Coniglio, Jeffery D. Lewis,Claudia Lang, Thomas G. Burns, Rabia Subhani-Siddique, Alan Weintraub, Howard Schub and E. Wayne Holden. A randomized, double-blind, placebo-controlled trial of single-dose intravenous secretin as treatment for children with autism. THE JOURNAL OF PEDIATRICS MAY 2001
